# The Humoral Immune Response Against the gB Vaccine: Lessons Learnt from Protection in Solid Organ Transplantation

**DOI:** 10.3390/vaccines7030067

**Published:** 2019-07-17

**Authors:** Ariane C. Gomes, Paul D. Griffiths, Matthew B. Reeves

**Affiliations:** Institute for Immunity and Transplantation, University College London, London NW3 2PF, UK

**Keywords:** cytomegalovirus, vaccine, antibodies, gB

## Abstract

Human cytomegalovirus (hCMV) is considered to be the highest priority for vaccine development. This view is underscored by the significant morbidity associated with congenital hCMV infection and viraemia in transplant patients. Although a number of vaccines have been trialed, none have been licensed. The hCMV vaccine candidate that has performed best in clinical trials to date is the recombinant glycoprotein B (gB) vaccine that has demonstrated protection, ranging from a 43% to 50% efficacy in three independent phase II trials. In this review, we focus on data from the phase II trial performed in solid organ transplant patients and the outcomes of follow-up studies attempting to identify immunological and mechanistic correlates of protection associated with this vaccine strategy. We relate this to other vaccine studies of gB as well as other vaccine strategies to determine areas of commonality and divergence. Finally, through the review, we discuss the unique challenges and opportunities presented with vaccine studies in transplant populations with recommendations that could empower subsequent trials.

## 1. Clinical Relevance of CMV

Human cytomegalovirus (hCMV) is a virus distributed worldwide, with reports estimating a prevalence ranging from 60% to 95% depending on factors, including age, ethnicity, and social economic status [1,2,3]. Transmission usually occurs via mucosal contact with bodily fluids, via the placenta, or following solid organ transplantation (SOT) from a seropositive donor into a seronegative or seropositive recipient. Following primary infection, reactivation of latent CMV or reinfection with a new strain in seropositive individuals may also result in clinical manifestations. For instance, after hematopoietic stem cell transplantation (HSCT), most cases of active infection are due to reactivation of latent infection [2]. The severity of hCMV infection depends largely on the immune status of the host, and around 90% of infected subjects are asymptomatic carriers [4]. In the immunocompetent, a primary infection usually results in limited morbidity progressing to lifelong latency with episodes of viral reactivation correlated to situations of immune stress. Indeed, viral reactivation has been described in cohorts of individuals with HIV infection, pregnancy, drug-induced immunosuppression for SOT, immune deficiency following HSCT, and the stresses associated with being an astronaut [5,6]. In many cases, hCMV infection is asymptomatic, but in some, it may progress to serious disease. hCMV is the number one cause of congenital infection with lifelong cognitive and hearing sequelae in the US [7] and has also been implicated as a contributor to allograft injury and rejection even in cases of subclinical viremia [8,9,10,11]. The development of effective antivirals has greatly reduced pathology in immunosuppressed SOT and HSCT recipients, however, the toxicity associated with antivirals, the development of resistance, and persistent long-term allograft dysfunction in spite of treatment indicates a need for alternative interventions [12,13]. In addition, previously unappreciated co-morbidities, such as reduced overall life-expectancy, increased mortality of patients admitted to the intensive care unit, and higher mortality in the elderly, are now being associated with chronic infection with hCMV [14].

Considering the economic and social burden associated with hCMV, especially for patients undergoing organ transplantation and neonates, the development of a vaccine targeting hCMV is considered a major priority by specialists and the Institute of Medicine since 2000 [15,16,17].

## 2. hCMV Immune Responses

hCMV has a complex interaction with the host with a long history of host/pathogen co-evolution that results in an infection with low morbidity in the immunocompetent and chronic infection instead of virus clearance [18]. Both cellular and humoral responses are important for viral control, and although the immune system is unable to clear the virus or offer complete protection against reinfection or reactivation, constant surveillance by the immune system is crucial to prevent progression to disease [19]. For instance, previous CMV infection reduces the congenital transmission from the high rate of 30% to 40% in seronegative to 1% to 2% in seropositive mothers [20,21]. Additionally, CMV risk-groups are generally composed of subjects experiencing immunosuppression or with an immature immune system, which indicates that the normal immune system is able to effectively prevent disease.

Both CD4^+^ and CD8^+^ T cell responses seem to be critical to the prevention of disease as evidenced by the effect of T cell immune suppression in transplants that results in the severe clinical manifestations of hCMV [22]. In a seropositive adult, it is estimated that up to 30% of the total pool of CD8^+^ T cells are responsive to CMV-derived peptides, the largest antigen-specific T cell population detected so far [23]. While the role of T cells in controlling the virus is a well-established consensus (reviewed elsewhere [24]), humoral responses have been less well studied. The remainder of this review will therefore focus on clinical data investigating the protection mediated by humoral responses, both natural and vaccine-induced responses, recent evidence from in vivo models, and the therapeutic feasibility of such approaches.

### Clinical Relevance of Humoral Immune Response

While T cells are thought to be crucial for the control of chronic infection [24], clinical data has provided evidence that humoral responses may offer protection against primary and acute infection, which may ultimately impact morbidity, transmission, and elimination of hCMV. Two randomized, placebo-controlled clinical trials in SOT provide important evidence on the matter by demonstrating a role for humoral responses in protection. Immunization with hCMV glycoprotein B (gB) vaccine plus MF59 adjuvant, a vaccine demonstrated to induce robust humoral responses, resulted in a 40% to 50% efficacy in SOT patients and the correlate of protection was the titer of antibodies made against gB [25]. To test the hypothesis of humoral protection in such patients, monoclonal antibodies against CMV were administered at the time of the transplant to seronegative transplant patients receiving organs from CMV seropositive donors and these significantly reduced CMV viremia post-transplant [26]. Specifically, it was shown that co-administration of two monoclonal antibodies, one against gH and the other against the UL131 component of the pentameric complex (PC) (gH/gL/UL128/UL130/UL131A), reduced the post-transplant incidence of CMV infection, delayed the detection of CMV viremia, and was associated with less CMV disease than in recipients of the placebo [26]. In support of this are studies performed with CMV-hyperimmune sera (CytoGam^®^). For example, administration of CytoGam has been suggested to improve the total survival and prevention of CMV-associated death in SOT recipients. Furthermore, progression to disease was significantly reduced in all patients receiving prophylactic CytoGam [27]. However, to date, these encouraging observations have not been supported with a randomized placebo-controlled trial.

A potential interpretation from trials in transplant patients is that considering the state of immunosuppression of such patients and that CTL responses are greatly reduced, the early control of the virus is likely to be mediated predominantly by other immune mechanisms, including antibodies. This in turn provides evidence that protection through vaccination may be an achievable goal, provided that appropriate humoral responses are induced. Most recently, an elegant study in a HSCT murine model demonstrated that reactivation of the virus was prevented by therapeutic administration of CMV positive immune sera. An important caveat of the study was that protection was only achieved whenever the virus strain of the donor and recipient was matched [28], posing an additional consideration when evaluating the efficacy of vaccines or other types of immunotherapy.

For congenital infection, pre-existing maternal immunity greatly reduces vertical transmission. Protection has been attributed to (i) the diversity of antigenic sites recognized in gH, (ii) level of in vitro neutralization, and (iii) CD4^+^ and CD8^+^ responses, including interleukin-2 production and lymphoproliferation [29,30]. Therapeutic interventions that attempted to reduce the incidence of the disease solely through humoral responses have failed to offer significant protection against fetal transmission, as for hyperimmune globulin tested in a randomized trial [31]. Higher levels and kinetics of IgG against the pentameric complex were correlated to reduced intrauterine transmission [32], and different patterns of avidity maturation as measured by the avidity index against total lysate of CMV were found amongst transmitting and non-transmitting mothers, where the fast development of high avidity responses was correlated to transmission while women with slower avidity maturation development were less prone to transmit the virus to the fetus [33]. Such findings may suggest that the pattern of IgG avidity maturation may influence transmission of the virus, and purely measuring total levels of anti-CMV IgG at a given time-point may not correlate with the efficacy of a vaccine. As evidence indicates, vertical transmission in natural infection seems to be controlled both by the humoral and cellular responses, posing an important consideration for vaccine design for this risk-group.

## 3. hCMV Vaccine

hCMV can cause disease either due to primary infection, secondary infection, or reactivation. As a result of that, both prophylactic and therapeutic vaccine approaches may be necessary to achieve protection across the different high-risk groups. A prophylactic vaccine able to prevent primary infection and transmission could be effective by inducing humoral responses, while therapeutic vaccines are likely to require the additional induction of potent T cell responses [34,35]. Consequently, candidates employing either strategy have been trialed in the past [36]. The clear progress achieved for the duration of the 50 years of attempts to develop an hCMV vaccine is tempered by the concomitant demonstration of the difficulties of targeting hCMV. One obstacle to the development of a vaccine against hCMV is that the virus causes major disease in immune-suppressed individuals with limited T cell function. Furthermore, it is clear that a previous infection does not grant unequivocal protection from re-infection [37]. This suggests that an effective vaccine for seropositives may need to “overwrite” previously established immune responses against hCMV that are not entirely protective [38]. Additionally, hCMV encodes numerous immune evasion mechanisms to evade many aspects of the immune response. Additionally, the virus can establish lifelong latency [19], possesses two modes of dissemination (cell-free and cell associated) that may require a distinct immune response to control them, as well as evidence of genomic variability [39], potentially requiring a vaccine that elicits heterologous protection.

In spite of such limitations, however, the hCMV vaccine field has developed substantially, resulting in the identification of several immunodominant antigens that are being harnessed in subunit vaccines [36,40,41].

### 3.1. Antigenic Targets of Humoral Responses

The hCMV genome encodes at least 165 canonical open reading frames [42], the largest viral genome that infects humans known so far. Of those, structural proteins, such as tegument proteins (e.g., pp65, pp150), glycoproteins, and non-structural proteins, such as immediate early protein 1 (IE1), are major targets for immune responses. Regarding humoral immunity, gB (encoded by gpUL55) and the gH-containing PC and trimeric complexes are the main targets of neutralizing antibodies [43,44]. Neutralizing antibodies directed against gB can block entry into most cell types whereas antibodies against the PC neutralize infection of epithelial, endothelial, and myeloid cells [43]. Viral gB is a conserved envelope glycoprotein involved with membrane fusion and cell entry in all cell types and is thus considered an important target for the development of humoral immunity. gB is encoded by a highly polymorphic gene and sequence analyses have identified clear differences between the gBs encoded by different hCMVs circulating in the population [45]. However, a recent study demonstrated that gB is generally highly conserved at the protein level with low antigenic variability [46]. An early study reported that 70% of the neutralizing activity in human sera could be absorbed by pre-treating the sera with gB prior to infection of human fibroblasts [47]. However, this contribution of anti-gB responses to neutralization has been challenged, with later studies reporting that up to 90% of sera-mediated neutralization in epithelial cells could be reversed by absorption with PC [32]. Monoclonal antibodies targeting the hCMV PC have also been shown to harbor potent neutralizing activity against hCMV infection in epithelial cells [43] and also prevented epithelial cell syncytium formation [48]. Of note, the rapidity with which antibodies against PC develop has been correlated with the level of protection to fetal transmission, thus arguing that PC antibody responses play an important protective role in vivo [32].

A number of potential practical and scientific concerns are associated with subunit vaccine production. For example, the production of recombinant full length envelope proteins, such as gB and gH, may be incompatible with large-scale production with a cost-effective process, which may turn a vaccine candidate economically and operationally unviable. Another aspect to consider is that for subunit vaccines utilizing either gB or gH, there is a reliance on the recombinant expression of truncated forms associated with adjuvants, or the use of viral vectors coding for such antigens. For the latter, a number of viral vector candidates have been tested with contrasting results, ranging from no humoral immunity to both neutralizing antibody and T cell responses. These illustrate the nature of the vector delivering the antigen is just as important as the antigen itself and is reviewed [40]. For the former, truncated or modified proteins tested to date have been unable to offer sufficient protection to achieve licensure. One possibility is that the truncation impacts on the correct induction of conformational antibodies and/or hide antigenic domains, reducing the efficacy of the vaccine against the target in the actual pathogen. Put simply, the end-point in any vaccine design process is not the generation of a potent response against the vaccine, but, of course, a vaccine-induced response that neutralizes hCMV infection. That said, it is likely that within the humoral response against viral proteins, there are specific responses that are protective and the identification of such a subset of responses against specific epitopes will be an important driver of future vaccine studies and may mitigate some of the issues associated with the production of full length recombinant proteins.

The identification of specific epitopes responsible for protective antibody responses will likely underpin future iterations of a gB based vaccine. Analyses of the natural humoral response to gB protein have established that at least five main antigenic domains (AD), named AD-1 to AD-5, exist. AD-1 corresponds to a domain of 80 amino acids positioned between residues 560 and 640 of gB (AD169 strain); AD-2 consists of two binding sites, one conserved among all the strains named site I (amino acids 50–54) and a variable region named site II (amino-acids 68–77); AD-3 is a linear epitope (amino acids 798–805) in the intraluminal region of gB; AD-5 is a discontinuous domain located between amino acids 133 and 343 (also known as domain I); and AD-4 is a discontinuous domain corresponding to amino acids 121–132 and 344–438 [49]. An important element of this characterization is the inference that responses against immuno-dominant domains may not contribute to protection [50]. Again, it is not necessarily a question of a large immune response but rather the right response. An aspect of this is the form in which the antigen is delivered. Consequently, enveloped virus-like particles (eVLPs) with the protein embedded in the envelope are currently being tested. It is hypothesized that the main advantage of such a strategy is that the protein may require less modification compared to the purified recombinant counterparts and currently, two eVLP-based vaccines against hCMV are currently under phase I evaluation [51,52].

Taken together, the current data in the hCMV vaccine development field shows that antigen optimization is imperative considering that the most immunogenic antigens are envelope glycoproteins.

### 3.2. The Drive Towards the Development of gB-Based Vaccines

The first attempts to develop a vaccine against hCMV were based on the traditional method of attenuated pathogens utilizing different hCMV strains, all of which would present native gB to the immune system. However, the Towne vaccine did not achieve significant protection based on the endpoint of the trial [53] and considering the risks of reversion to virulence, this strategy was abandoned in favor of recombinant subunit vaccines [40]. Consequently, different approaches and platforms have been tested against hCMV. Candidates harnessing technologies, such as live-attenuated, recombinant sub-unit vaccine, eVLPs, replication-impaired hCMV, nucleic acid vaccine, and viral vectors, including disabled infection single cycle (DISC) vectors, have been developed (for a detailed review in hCMV vaccines, see [35,36]). Of those, the candidate that has shown the greatest promise thus far in clinical trials is the gB/MF59 vaccine and will become the focus of this review.

### 3.3. gB/MF59 Vaccine

The gB/MF59 vaccine is a recombinant vaccine formulated with a modified form of the gB protein in combination with the microfluidized adjuvant 59 (MF59, Fluad^®^ Novartis), a squalene-based emulsion [54]. This candidate developed by Sanofi is currently the most well characterized vaccine candidate, trialed in several phase I and three II trials in different high-risk populations. The immunization schedule included three doses of the vaccine given at 0, 1, and 6 months and the outcomes varied according to the relevant clinical manifestation of hCMV in the groups investigated.

The first phase II trial tested the efficacy of the vaccine in a population of hCMV-seronegative women immunized in the postpartum period [55]. The efficacy of the vaccine as a prophylactic intervention to prevent CMV acquisition was 50% and the chosen endpoint was the time until detection of primary infection during a 42-month follow up. In follow-up studies of the phase II postpartum vaccinees, it was also demonstrated that whilst gB/MF59 had minimal impact in the in vivo replication of the virus, the seroconverted vaccinees had reduced viral shedding in the saliva by an order of magnitude comparable to the placebo [56]. These intriguing data could have important implications for long term control of hCMV transmission, an important criterion for a successful hCMV vaccine.

The second phase II trial followed the same vaccination regimen and the same end-point in a population of seronegative adolescent girls. The efficacy was similar, with a 43% reduction in hCMV acquisition although the results failed to achieve statistical significance [57]. The third phase II trial evaluated the efficacy in both populations of seronegative and seropositive patients awaiting SOT and demonstrated levels of protection similar to that observed in the original Pass study [48], although it is important to note that protection only reached statistical significance in the seronegative recipients receiving an organ from a seropositive donor. However, vaccination correlated with reduced viremia and reduced duration of antiviral treatment. Positive outcomes were correlated with the magnitude of anti-gB humoral responses; in the sub-group that developed viremia anti-gB, antibody titers were inversely correlated with the duration of viremia [25]. Importantly, the gB vaccine was demonstrated to induce high levels of anti-gB antibodies in seronegatives and also, to boost pre-existing levels of gB antibodies in the seropositives.

### 3.4. gB/MF59 Vaccine and Correlates Protection

An important aspect of the follow up studies of the respective vaccine trial sera is the identification of correlates of protection. Surprisingly, it was demonstrated that the sera from immunized seronegative patients from two trials did not promote virus neutralization in fibroblasts despite the positive outcomes promoted by the vaccine discussed earlier [58,59]. Furthermore, protection could not be explained by antibody-dependent cellular cytotoxicity (ADCC) although Nelson et al. demonstrated that antibody dependent cellular phagocytosis (ADCP) was increased in the vaccinated group of postpartum women [58] and that subsequent phagocytosis resulted in abortive infection of myeloid cells. Moreover, the genotyping of the hCMV infecting gB/MF59 vaccine recipients demonstrated that acquisition of genetically related gB genotypes was reduced in the vaccinees compared to the placebo, suggesting that the gB genotype may be important [56]. However, a recent study by Foglierini et al. investigated the genomic polymorphism in the sequence of gB in 207 strains, including the variant included in the gB/MF59 vaccine. Sequence and antigenic analysis demonstrated that despite gB being highly polymorphic [45], protein and antigenic hotspots are highly conserved across vaccine variants, circulating, and laboratory strains, suggesting that the reduced protection conferred by the vaccine was not due to polymorphism on gB [46] as previously speculated.

An alternative explanation is that the vaccine is inducing a response to a novel epitope that is distinct from the classic ADs described for gB. Indeed, analysis of the sera seronegative cohort from the SOT study argued that the gB humoral response was not directed to the known Ads, including AD1, AD2, AD4, and AD5 [59]. Furthermore, the study of the seronegative postpartum cohort revealed that the vaccinees had a response dominated by antibodies targeting AD3 (76% of total). This was in contrast to the AD3 response seen in healthy hCMV seropositive controls, where anti-AD3 antibodies account for 32% of the total gB response. Collectively, the results from these recent studies provide evidence that the humoral responses mounted against the vaccine gB and gB from natural infections are distinct, requiring careful considerations of this effect in future studies. All told, it is fair to say that the precise mechanism through which the gB/MF59 offers protection to vaccinated seronegative individuals remains a matter of investigation [60].

The SOT study allowed for the additional study of the impact of vaccination on the humoral response against gB in seropositive individuals. The analysis of the humoral response in the vaccinated SOT patients against the known ADs from gB suggested that the vaccine predominantly boosted previous AD-responses already present in the seropositive cohort [61]. This was overtly observed with responses against AD2 [52], a subdominant epitope that induces antibody responses in only 50% of infected individuals [62]. Specifically, seropositive patients who already had AD2 responses had a significant boost in the anti-AD2 IgG levels, but AD2 negative seropositive patients remained AD2-negative following immunization, consistent with the failure of the vaccine to induce detectable AD2 responses in the seronegative SOT cohort. Taken together, this suggests the phenomenon of original antigenic sin may influence the outcome of immunization in seropositive individuals. It was demonstrated that those with better outcomes (e.g., reduced viremia and duration of antiviral treatment) had higher levels of antibodies against AD2 [63], thus overcoming the potential impact of antigenic sin becomes important if only a subset of infected individuals make a protective response against gB prior to vaccination.

### 3.5. Clinical Trial Endpoints and Correlates of Protection for hCMV Vaccine

Classically, the detection of neutralizing antibodies is one of the major correlates of protection for vaccine evaluation. The lack of evidence for the neutralizing activity of sera from the two gB/MF59 clinical trials previously discussed therefore challenges the validity of using this concept to assess hCMV vaccines based on gB, or at least ensures we reconsider our criteria (and in vitro experimental approaches) for evaluating future hCMV vaccines. The low neutralization titers could be a result of either the inability of current in vitro neutralization tests to reflect neutralization capacity in vivo or that the vaccine is inducing protection via non-neutralizing mechanisms. Supporting the former, a study conducted by Bootz et al. tested how the in vitro neutralization activity of mAb correlated with the therapeutic activity of the mAbs in vivo. A panel of mAbs against gB was generated and ranked according to a neutralization score. Then, RAG-/-mice, a strain deficient in functional B and T cells and depleted of NK cells, were challenged with a lethal dose of murine CMV (mCMV), and the efficacy of both non-neutralizing and neutralizing mAb were tested in protecting animals against lethality. Both neutralizing and non-neutralizing mAbs conferred protection and the efficacy was correlated with the half-life of the antibody in the organism and not the results from in vitro neutralization assays [64]. In line with the latter, results from Nelson et al. found that sera from immunized subjects increased ADCP of the hCMV virion and that following phagocytosis, the resulting infection was not productive [58]. Indeed, it is interesting to highlight that infection of macrophages with antibody coated hCMV resulted in abortive infection, activation of the antiviral state through an IFN-independent mechanism, and antigen presentation to T cells [65], which may explain the efficacy of the vaccine in the absence of strong neutralizing responses. Furthermore, in mice, measuring the control of the cell-to-cell spread of the virus was a better correlate of clinical outcome of mCMV activation and mortality following HSCT compared to classical neutralization assays [28]. More generally, this reiterates the importance of evaluating the current tests, such as virus neutralization, as a correlate of hCMV vaccine efficacy to ensure we are not unwittingly hindering vaccine development and evaluation by assessing with inadequate methods.

In addition to correlates of protection, endpoints for clinical trial had been extensively debated. Traditionally, endpoints are sero-conversion, systemic replication, and development of CMV-associated disease [41]. Such correlates, however, may vary across the different groups affected by the virus and some correlates, such as virus acquisition, are an unrealistic outcome to measure considering the low R_o_ of the virus [17]. It has been estimated that to achieve enough statistical power in a phase III trial, considering a vaccine with 50% efficiency, at least 50,000 volunteers would need to be recruited [4]. Studies focused on congenital infection have chosen different endpoints in the past, such as intra-uterine transmission, development of cCMV syndrome, or virus shedding. Hoping to address the disparities in the trials that may be hindering progress, guidelines for phase III clinical trial endpoints were proposed in 2013 and can be reviewed in here [41].

Clinical trials with patients undergoing SOT provide an excellent model to investigate the pathology of the virus and efficacy of vaccines. This unique population provides the closest example of a human challenge model with hCMV that does not invoke major ethical considerations and overcomes most of the difficulties in hCMV clinical trials mentioned above. First, Atabani et al. reported that around 50% of organs available for transplant were hCMV seropositive and, of those, 78% of the transplants resulted in hCMV transmission to seronegative recipients [66]. With high transmission rates, fewer participants are required to power the study. Some SOT patients will be experiencing either primary infection (78% viraemia) when receiving a transplant from a seropositive donor, reactivation of the latent virus in seropositives due to immunosuppression (40% viraemia), or yet re-challenge a previously seropositive recipient of an organ from a seropositive donor plus the risk of reactivation (54% viraemia). Furthermore, the time of viral challenge is known (i.e., time of transplant). All this taken together allows access to information unavailable in other populations and could contribute to understanding the pathology and immune responses against this complex pathogen. In addition to that, the model provides clear measurable outcomes, such as the onset and duration of viremia, rendering efficacy studies easier to perform and interpret. This model is especially valuable to investigate protection mediated by antibodies, considering that transplant recipients will be experiencing drug-induced immuno-suppression, reducing the role of T cells in protection in this setting. It is important to accept, however, that there are caveats to using this as a simple model of infection. Most pertinently, a major difference is that the acquisition of CMV during organ transplant is clearly different from natural infection, bypassing mucosal and epithelial contact, which may impact the immune response and control during the initial stages of infection. In addition, the strain of hCMV and the dose transferred from the donor to the recipient cannot be controlled.

### 3.6. gB-Based Vaccines and Beyond

The failure of gB-based vaccines to achieve sufficient levels of protection to progress to licensing has re-focused attempts to develop an hCMV vaccine against different targets for antibody responses or associating gB with T cell epitopes through viral vectors (Table 1). That said, the recent results analyzing gB humoral responses in phase II trials may pave the way to the reinvestigation of gB vaccines as candidates if responses against novel epitopes can be identified in the gB vaccine responses. To facilitate this process, important lessons may be drawn from years of attempts and failures to develop a peptide vaccine against other pathogens.

It is agreed in the vaccine field that to achieve protection via antibodies, an ideal candidate should induce high levels of class switched antigen-specific antibodies, with high-affinity, and ideally inducing the formation of memory plasma cells [67]. In addition to providing protection, a vaccine must be well tolerated and safe. Subunit vaccines are often safer in comparison to the attenuated pathogen counterparts, however, isolated proteins usually fail to induce the full repertoire of immune responses. This in itself may not be a problem—all that matters is that the immune response is sufficiently protective. Thus, selection of the ideal antigen is as important as the choice of adjuvants in order to present it in a relevant manner to the immune system [68].

Following the identification of antigenic domains from gB, a logical step is to develop subunit vaccines with the more protective ADs without the other confounding epitopes. It has been estimated that >90% of the humoral responses against gB in natural infection are not neutralizing [49]. Humoral responses against gB are dominated by antibodies targeting AD1, present in nearly all seropositive sera [69]. In respect to the remainder ADs, AD2 is present in nearly 50% of screened seropositives, AD3 in 37% [58], AD4 in >90%, and AD5 in >50% [49]. Of those, AD2 was correlated to better outcomes in transplant patients [63]. This may suggest that to overcome the effect of original antigenic sin [61], a gB sub-unit vaccine containing only AD2 (or another protective epitope) may be more advantageous. Such an approach has been tested by associating AD2 with a carrier protein, CRM197 [70]. Preclinical evaluation of the vaccine demonstrated that the vaccine induced antigen-specific antibody responses, however, such antibodies did not show any neutralizing activity against the virus, leaving room for further developments in the design of the vaccine. However, mindful of recent studies of humoral responses against the gB vaccine in vivo, with particular reference to a failure to identify evidence of a neutralizing response, may not necessarily mean this vaccine is ineffective. This can only be effectively assessed in a phase II challenge trial. In addition to that, preclinical development of an AD-2–specific therapeutic mAb is currently being tested [71], indicating that gB-derived epitopes are likely to be one prominent direction for vaccine development.

An important and sometimes marginalized aspect of vaccination is the choice of adjuvant or vaccine platform. At this stage, what has been learned about the activation process of B cells can be readily translated to a rational vaccine design. Appropriate activation of B cells that bypasses the need for T cell help should include (i) multiple B cell receptor (BCR) crosslinking, (ii) efficient draining of antigens to lymph nodes to facilitate the encountering of antigen by B cells, and (iii) activation of pattern recognition receptors (PRRs) [72]. First, it has been demonstrated that the repetitiveness of a protein is a pathogen associated structural pattern (PASP) that activates B cells [73]. Repetitive structures exposing antigens in an organized fashion is a characteristic of pathogens, such as viruses, that rely on one or two monomeric subunits in a quasi-equivalent conformation to form a capsid. This level of repetition is absent in vertebrates and allows deposition of complement components and crosslinking of several BCRs in one single cell, providing a strong activation signal even in the absence of other activation signals [73]. This approach has been extensively explored by associating peptides to carrier proteins with multiple conjugation sites, nanoparticles, and virus-like particles (VLPs), which imprints a viral fingerprint to small and non-immunogenic sequences, such as peptides. An additional lesson learned from the activation of B cells and other APCs is that the size of particulate proteins influences the draining of particles through the lymphatic system and overall immunogenicity [74,75,76]. Particles that fall within the size range of 10 to 200 nm are able to flow freely from the periphery and reach lymph nodes, including the sub-capsular sinus and B cell areas, where relevant cells of the immune system reside and initiate adaptive immunity [77]. Smaller particles, such as peptides, do not drain from the periphery and are mostly degraded without eliciting immune responses; larger particles rely on cell-associated transport, which may limit the cell types that get activated [78]. Thus, adjusting the size of sub-unit antigens (e.g., by associating antigens with carrier proteins) is an important consideration during vaccine design that greatly impacts immunogenicity. Another important consideration is that proteins and particulate antigens are often non-immunogenic because of the inability to offer a pathogen-associated molecular pattern (PAMP), thus failing to activate immune cells. B cells are equipped with several PRRs that sense PAMPs and result in proliferation and differentiation in plasma cells, and germinal center formation [79], which supports the generation of neutralizing antibodies and protective humoral immunity [80]. In addition to the activation and differentiation of B cells, TLR ligands, especially TLR9 and TLR7, associated with antigens promote antigen-specific responses with class-switched antibodies even with a single immunization [81,82,83].

In summary, by combining the current knowledge of antigenic domains derived from hCMV and the requirements for appropriate B cell activation, it may be possible to develop hCMV vaccines with a higher immunogenicity that are able to induce not only high levels of antibodies, but also the protective type of humoral responses. In addition, a close evaluation of the current tests and correlates of the protection and vaccine efficacy in place should be also be revisited.

## 4. Conclusions

The clinical data so far has demonstrated that antibody-inducing vaccines and mAbs have improved the outcomes of subjects in high risk groups. Additionally, the observation by Nelson et al. that seroconverted gB/MF59 vaccine recipients have similar viral replication but reduced viral shedding in saliva may indicate that humoral responses may impact the transmission of hCMV, and those findings will also help to shape the directions of the development of vaccines that induce humoral responses, which may shift from prophylactic vaccines to transmission-blocking strategies promoting herd immunity. Moreover, data have provided important lessons about immune responses against CMV that will surely contribute to the development of an effective vaccine. The next vaccine designs should thoughtfully consider characteristics of the virus, such as optimal antigenic targets, including the induction of cross-protection across the different hCMV strains, and appropriate correlates of protection and efficacy.

## Figures and Tables

**Table 1:** Recent clinical trials in hCMV vaccines in transplant patients.

Vaccine	Response	Condition	Phase	URL
Active trials				
HB-101 bivalent viral vector vaccine	Antibodies to gB, T cells to pp65	SOT	2	NCT03629080
CMV-MVA Triplex Vaccine	T cells to pp65, E1-exon4, IE2-exon5	HSCT	1 & 2	NCT03354728
CMV-MVA Triplex Vaccine	T cells to pp65, E1-exon4, IE2-exon5	HSCT	2	NCT03560752
BD03—trivalent DNA vaccine		SOT	1	NCT03576014
CMV-MVA Triplex Vaccine	T cells to pp65, E1-exon4, IE2-exon5	HSCT	1	NCT03383055
Completed trials				
ASP0113—bivalent DNA vaccine	Antibodies to gB, T cells to pp65	HSCT	3	NCT01877655
ASP0113—bivalent DNA vaccine	Antibodies to gB, T cells to pp65	SOT	2	NCT01974206
CMV gB vaccine	Antibodies to gB	SOT	2	NCT00299260
ALVAC-CMV (vCP260)	T cells to pp65	HSCT	2	NCT00353977
VCL-CB01—Bivalent DNA vaccine	Antibodies to gB, T cells to pp65	HSCT	2	NCT00285259
CMV-MVA Triplex Vaccine	T cells to pp65, E1-exon4, IE2-exon5	HSCT	2	NCT02506933
tetanus-CMV fusion peptide vaccine	T cells to pp65	HSCT	1	NCT01588015

CMV: cytomegalovirus; MVA: modified Vaccinia Ankara; gB: glycoprotein B; pp65: phosphoprotein 65; HSCT: haematopoietic stem cell transplant; SOT: solid organ transplant.

## References

[1] Zuhair M., Smit G.S.A., Wallis G., Jabbar F., Smith C., Devleesschauwer B., Griffiths P. (2019). Estimation of the worldwide seroprevalence of cytomegalovirus: A systematic review and meta-analysis. Rev. Med. Virol..

[2] Manicklal S., Emery V.C., Lazzarotto T., Boppana S.B., Gupta R.K. (2013). The “Silent” global burden of congenital cytomegalovirus. Clin. Microbiol. Rev..

[3] Panagou E., Zakout G., Keshani J., Smith C., Irish D., Mackinnon S., Kottaridis P., Fielding A., Griffiths P.D. (2016). Cytomegalovirus pre-emptive therapy after hematopoietic stem cell transplantation in the era of real-time quantitative PCR: comparison with recipients of solid organ transplants. Transpl. Infect. Dis..

[4] Adler S.P. (2013). Immunization to prevent congenital cytomegalovirus infection. Br. Med. Bull..

[5] Mehta S.K., Crucian B.E., Stowe R.P., Simpson R.J., Ott C.M., Sams C.F., Pierson D.L. (2013). Reactivation of latent viruses is associated with increased plasma cytokines in astronauts. Cytokine.

[6] Barbosa N., Yamamoto A.Y., Duarte G., Aragon D.C., Fowler K.B., Boppana S., Britt W.J., Mussi-Pinhata M. (2018). Cytomegalovirus (CMV) shedding in seropositive pregnant women from a high prevalence population: “The Brazilian Cytomegalovirus Hearing and Maternal Secondary Infection Study (BraCHS)”. Infect. Dis. Soc. Am..

[7] Fowler K.B., Stagno S., Pass R.F. (2003). Maternal Immunity and Prevention of Congenital Cytomegalovirus Infection. J. Am. Med. Assoc..

[8] Legendre C., Pascual M. (2008). Improving Outcomes for Solid-Organ Transplant Recipients At Risk from Cytomegalovirus Infection: Late-Onset Disease and Indirect Consequences. Clin. Infect. Dis..

[9] Smith J.M., Corey L., Bittner R., Finn L.S., Healey P.J., Davis C.L., McDonald R.A. (2010). Subclinical Viremia Increases Risk for Chronic Allograft Injury in Pediatric Renal Transplantation. J. Am. Soc. Nephrol..

[10] Green M.L., Leisenring W., Xie H., Mast T.C., Cui Y., Sandmaier B.M., Sorror M.L., Goyal S., Özkök S., Yi J. (2016). Cytomegalovirus viral load and mortality after haemopoietic stem cell transplantation in the era of pre-emptive therapy: a retrospective cohort study. Lancet Haematol..

[11] Chan S.T., Logan A.C. (2017). The clinical impact of cytomegalovirus infection following allogeneic hematopoietic cell transplantation: Why the quest for meaningful prophylaxis still matters. Blood Rev..

[12] Tang J. (2013). Cytomegaloviruses: From Molecular Pathogenesis to Intervention. Emerg. Infect. Dis..

[13] Chemaly R.F., Ullmann A.J., Stoelben S., Richard M.P., Bornhäuser M., Groth C., Einsele H., Silverman M., Mullane K.M., Brown J. (2014). Letermovir for Cytomegalovirus Prophylaxis in Hematopoietic-Cell Transplantation. N. Engl. J. Med..

[14] Simanek A.M., Dowd J.B., Pawelec G., Melzer D., Dutta A., Aiello A.E. (2011). Seropositivity to cytomegalovirus, inflammation, all-cause and cardiovascular disease-related mortality in the United States. PLoS ONE.

[15] Stratton K.R., Durch J.S., Robert S. (2000). Vaccines for the 21st Century.

[16] Modlin J.F., Arvin A.M., Fast P., Myers M., Plotkin S., Rabinovich R. (2004). Vaccine Development to Prevent Cytomegalovirus Disease: Report from the National Vaccine Advisory Committee. Clin. Infect. Dis..

[17] Griffiths P.D., McLean A., Emery V.C. (2001). Encouraging prospects for immunisation against primary cytomegalovirus infection. Vaccine.

[18] Poole E., Dallas S.R.M.G., Colston J., Joseph R.S.V., Sinclair J. (2011). Virally induced changes in cellular microRNAs maintain latency of human cytomegalovirus in CD34+ progenitors. J. Gen. Virol..

[19] Murray M., Peters N., Reeves M. (2018). Navigating the Host Cell Response during Entry into Sites of Latent Cytomegalovirus Infection. Pathogens.

[20] Doorbar J., Egawa N., Griffin H., Kranjec C., Murakami I. (2015). Review and meta-analysis of the epidemiology of congenital cytomegalovirus (CMV) infection. Rev. Med. Virol..

[21] Gerna G., Lilleri D. (2019). Human cytomegalovirus (HCMV) infection/re-infection: Development of a protective HCMV vaccine. New Microbiol..

[22] Reddehase M.J. (2002). Antigens and immunoevasins: Opponents in cytomegalovirus immune surveillance. Nat. Rev. Immunol..

[23] Sylwester A.W., Mitchell B.L., Edgar J.B., Taormina C., Pelte C., Ruchti F., Sleath P.R., Grabstein K.H., Hosken N.A., Kern F. (2005). Broadly targeted human cytomegalovirus-specific CD4+ and CD8+ T cells dominate the memory compartments of exposed subjects. J. Exp. Med..

[24] Klenerman P., Oxenius A. (2016). T cell responses to cytomegalovirus. Nat. Publ. Gr..

[25] Griffiths P.D., Stanton A., McCarrell E., Smith C., Osman M., Harber M., Davenport A., Jones G., Wheeler D.C., OBeirne J. (2011). Cytomegalovirus glycoprotein-B vaccine with MF59 adjuvant in transplant recipients: A phase 2 randomised placebo-controlled trial. Lancet.

[26] Ishida J.H., Patel A., Mehta A.K., Gatault P., Mcbride J.M., Burgess T., Derby M.A., Snydman D.R., Emu B., Feierbach B. (2017). Phase 2 randomized, double-blind, placebo-controlled trial of RG7667, a combination monoclonal antibody, for prevention of Cytomegalovirus Infection in high-risk kidney transplant. Antimicrob. Agents Chemother..

[27] Bonaros N., Mayer B., Schachner T., Laufer G., Kocher A. (2008). CMV-hyperimmune globulin for preventing cytomegalovirus infection and disease in solid organ transplant recipients: A meta-analysis. Clin. Transplant..

[28] Martins J.P., Andoniou C.E., Fleming P., Kuns R.D., Schuster I.S., Voigt V., Daly S., Varelias A., Tey S., Degli-esposti M.A. (2019). strain-specific antibody therapy prevents cytomegalovirus reactivation after transplantation. Science.

[29] Lilleri D., Gerna G. (2017). Maternal immune correlates of protection from human cytomegalovirus transmission to the fetus after primary infection in pregnancy. Rev. Med. Virol..

[30] Revello M.G., Lilleri D., Zavattoni M., Furione M., Genini E., Comolli G., Gerna G. (2005). Lymphoproliferative Response in Primary Human Cytomegalovirus (HCMV) Infection Is Delayed in HCMV Transmitter Mothers. J. Infect. Dis..

[31] Revello M.G., Lazzarotto T., Guerra B., Spinillo A., Ferrazzi E., Kustermann A., Guaschino S., Vergani P., Todros T., Frusca T. (2014). A randomized trial of hyperimmune globulin to prevent congenital cytomegalovirus. Obstet. Gynecol. Surv..

[32] Lilleri D., Kabanova A., Revello M.G., Percivalle E., Sarasini A., Genini E., Sallusto F., Lanzavecchia A., Corti D., Gerna G. (2013). Fetal Human Cytomegalovirus Transmission Correlates with Delayed Maternal Antibodies to gH/gL/pUL128-130-131 Complex during Primary Infection. PLoS ONE.

[33] Boppana S.B., Britt W.J. (1995). Antiviral antibody responses and intrauterine transmission after primary maternal cytomegalovirus infection. J. Infect. Dis..

[34] Plotkin S.A., Boppana S.B. (2018). Vaccination against the human cytomegalovirus. Vaccine.

[35] Anderholm K.M., Bierle C.J., Schleiss M.R. (2016). Cytomegalovirus Vaccines: Current Status and Future Prospects. Drugs.

[36] Schleiss M.R. (2016). Cytomegalovirus vaccines under clinical development. J. Virus Erad..

[37] Ross S.A., Arora N., Novak Z., Fowler K.B., Britt W.J., Boppana S.B. (2009). Cytomegalovirus Reinfections in Healthy Seroimmune Women. J. Infect. Dis..

[38] La Rosa C., Diamond D.J. (2012). The immune response to human CMV. Future Virol..

[39] Wilkinson G.W.G., Davison A.J., Tomasec P., Fielding C.A., Aicheler R., Murrell I., Seirafian S., Wang E.C.Y., Weekes M., Lehner P.J. (2015). Human cytomegalovirus: taking the strain. Med. Microbiol. Immunol..

[40] Schleiss M.R., Permar S.R., Plotkin S.A. (2017). Progress toward Development of a Vaccine against Congenital Cytomegalovirus Infection. Clin. Vaccine Immunol..

[41] Krause P.R., Bialek S.R., Boppana S.B., Griffiths P.D., Laughlin C.A., Ljungman P., Mocarski E.S., Pass R.F., Read J.S., Schleiss M.R. (2013). Priorities for CMV vaccine development. Vaccine.

[42] Murphy E., Shenk T.E., Shenk T.E., Stinski M.F. (2008). Human Cytomegalovirus Genome. Human Cytomegalovirus. Current Topics in Microbiology and Immunology.

[43] Fouts A.E., Chan P., Stephan J.-P., Vandlen R., Feierbach B. (2012). Antibodies against the gH/gL/UL128/UL130/UL131 Complex Comprise the Majority of the Anti-Cytomegalovirus (Anti-CMV) Neutralizing Antibody Response in CMV Hyperimmune Globulin. J. Virol..

[44] Wussow F., Chiuppesi F., Contreras H., Diamond D. (2017). Neutralization of Human Cytomegalovirus Entry into Fibroblasts and Epithelial Cells. Vaccines.

[45] Renzette N., Bhattacharjee B., Jensen J.D., Gibson L., Kowalik T.F. (2011). Extensive Genome-Wide Variability of Human Cytomegalovirus in Congenitally Infected Infants. PLoS Pathog..

[46] Foglierini M., Marcandalli J., Perez L. (2019). HCMV Envelope Glycoprotein Diversity Demystified. Front. Microbiol..

[47] Britt W.J., Vugler L., Butfiloski E.J., Stephens E.B. (1990). Cell Surface Expression of Human Cytomegalovirus (HCMV) gp55-116 (gB): Use of HCMV-Recombinant Vaccinia Virus-Infected Cells in Analysis of the Human Neutralizing Antibody Response. J. Virol..

[48] Gerna G., Percivalle E., Perez L., Lanzavecchia A., Lilleri D. (2016). Monoclonal Antibodies to Different Components of the Human Cytomegalovirus (HCMV) Pentamer gH/gL/pUL128L and Trimer gH/gL/gO as well as Antibodies Elicited during Primary HCMV Infection Prevent Epithelial Cell Syncytium Formation. J. Virol..

[49] Pötzsch S., Spindler N., Wiegers A.K., Fisch T., Rücker P., Sticht H., Grieb N., Baroti T., Weisel F., Stamminger T. (2011). B cell repertoire analysis identifies new antigenic domains on glycoprotein b of human cytomegalovirus which are target of neutralizing antibodies. PLoS Pathog..

[50] Spindler N., Rucker P., Potzsch S., Diestel U., Sticht H., Martin-Parras L., Winkler T.H., Mach M. (2013). Characterization of a Discontinuous Neutralizing Epitope on Glycoprotein B of Human Cytomegalovirus. J. Virol..

[51] Kirchmeier M., Fluckiger A.C., Soare C., Bozic J., Ontsouka B., Ahmed T., Diress A., Pereira L., Schodel F., Plotkin S. (2014). Enveloped virus-like particle expression of human cytomegalovirus glycoprotein B antigen induces antibodies with potent and broad neutralizing activity. Clin. Vaccine Immunol..

[52] Vicente T., Burri S., Wellnitz S., Walsh K., Rothe S., Liderfelt J. (2014). Fully aseptic single-use cross flow filtration system for clarification and concentration of cytomegalovirus-like particles. Eng. Life Sci..

[53] Plotkin S.A., Friedman H.M., Fleisher G.R., Dafoe D.C., Grossman R.A., Lynn Smiley M., Starr S.E., Wlodaver C., Friedman A.D., Barker C.F. (1984). Towne-Vaccine-Induced Prevention of Cytomegalovirus Disease After Renal Transplants. Lancet.

[54] Pass R.F., Duliegè A., Boppana S., Sekulovich R., Percell S., Britt W., Burke R.L. (2002). A Subunit Cytomegalovirus Vaccine Based on Recombinant Envelope Glycoprotein B and a New Adjuvant. J. Infect. Dis..

[55] Pass R.F., Zhang C., Evans A., Simpson T., Huang M., Andrews W., Corey L., Hill J., Davis E., Flanigan C. (2010). Vaccine prevention of maternal CMV infection. N. Engl. J. Med..

[56] Nelson C.S., Vera Cruz D., Su M., Xie G., Vandergrift N., Pass R.F., Forman M., Diener-West M., Koelle K., Arav-Boger R. (2018). Intrahost Dynamics of Human Cytomegalovirus Variants Acquired by Seronegative Glycoprotein B Vaccinees. J. Virol..

[57] Bernstein D.I., Munoz F.M., Callahan S.T., Rupp R., Wootton S.H., Edwards K.M., Turley C.B., Stanberry L.R., Patel S.M., Mcneal M.M. (2016). Safety and efficacy of a cytomegalovirus glycoprotein B (gB) vaccine in adolescent girls: A randomized clinical trial. Vaccine.

[58] Nelson C.S., Huffman T., Jenks J.A., Cisneros de la Rosa E., Xie G., Vandergrift N., Pass R.F., Pollara J., Permar S.R., Pass R.F. (2018). HCMV glycoprotein B subunit vaccine efficacy mediated by nonneutralizing antibody effector functions. Proc. Natl. Acad. Sci. USA.

[59] Baraniak I., Kropff B., Ambrose L., McIntosh M., McLean G.R., Pichon S., Atkinson C., Milne R.S.B., Mach M., Griffiths P.D. (2018). Protection from cytomegalovirus viremia following glycoprotein B vaccination is not dependent on neutralizing antibodies. Proc. Natl. Acad. Sci. USA.

[60] Schleiss M.R. (2018). Recombinant cytomegalovirus glycoprotein B vaccine: Rethinking the immunological basis of protection. Proc. Natl. Acad. Sci. USA.

[61] Baraniak I., Kern F., Holenya P., Griffiths P., Reeves M. (2019). Original antigenic sin shapes the immunological repertoire evoked by HCMV gB-MF59 vaccine in seropositive recipients. J. Infect. Dis..

[62] Lantto J., Fletcher J.M., Ohlin M. (2003). Binding characteristics determine the neutralizing potential of antibody fragments specific for antigenic domain 2 on glycoprotein b of human cytomegalovirus. Virology.

[63] Baraniak I., Kropff B., Mclean G.R., Pichon S., Piras-douce F., Milne R.S.B., Smith C., Mach M., Griffiths P.D., Reeves M.B. (2018). Epitope-Specific Humoral Responses to Human Cytomegalovirus Glycoprotein-B Vaccine with MF59: Anti-AD2 Levels Correlate With Protection From Viremia. J. Infect. Dis..

[64] Bootz A., Karbach A., Spindler J., Kropff B., Reuter N., Sticht H., Winkler T.H., Britt W.J., Mach M. (2017). Protective capacity of neutralizing and non-neutralizing antibodies against glycoprotein B of cytomegalovirus. PLoS Pathog..

[65] Wu Z., Qin R., Wang L., Bosso M., Scherer M., Stamminger T., Hotter D., Mertens T., Frascaroli G. (2017). Human Cytomegalovirus Particles Treated with Specific Antibodies Induce Intrinsic and Adaptive but Not Innate Immune Responses. J. Virol..

[66] Atabani S.F., Smith C., Atkinson C., Aldridge R.W., Rodriguez-Perálvarez M., Rolando N., Harber M., Jones G., O’Riordan A., Burroughs A.K. (2012). Cytomegalovirus replication kinetics in solid organ transplant recipients managed by preemptive therapy. Am. J. Transplant..

[67] Plotkin S.A., Plotkin S.L. (2011). The development of vaccines: how the past led to the future. Nat. Rev. Microbiol..

[68] Roldão A., Mellado M.C.M., Castilho L.R., Carrondo M.J.T., Alves P.M. (2010). Virus-like particles in vaccine development. Expert Rev. Vaccines.

[69] Schoppel K., Haßfurther E., Britt W., Ohlin M., Borrebaeck C.A.K., Mach M. (1996). Antibodies specific for the antigenic domain 1 of glycoprotein B (gpUL55) of human cytomegalovirus bind to different substructures. Virology.

[70] Finnefrock A.C., Freed D.C., Tang A., Li F., He X., Wu C., Nahas D., Wang D., Fu T.M. (2016). Preclinical evaluations of peptide-conjugate vaccines targeting the antigenic domain-2 of glycoprotein B of human cytomegalovirus. Hum. Vaccines Immunother..

[71] Kauvar L.M., Liu K., Park M., DeChene N., Stephenson R., Tenorio E., Ellsworth S.L., Tabata T., Petitt M., Tsuge M. (2015). A high-affinity native human antibody neutralizes human cytomegalovirus infection of diverse cell types. Antimicrob. Agents Chemother..

[72] Batista F.D., Harwood N.E. (2009). The who, how and where of antigen presentation to B cells. Nat. Rev. Immunol..

[73] Zinkernagel R.M. (2003). On natural and artificial vaccinations. Annu. Rev. Immunol..

[74] Reddy S.T., van der Vlies A.J., Simeoni E., O’Neil C.P., Swartz M.A., Hubbell J.A. (2007). Exploiting lymphatic transport and complement activation in nanoparticle vaccines. Nat. Biotechnol..

[75] Gomes A.C., Flace A., Saudan P., Zabel F., Cabral-Miranda G., Turabi A.E., Manolova V., Bachmann M.F. (2017). Adjusted Particle Size Eliminates the Need of Linkage of Antigen and Adjuvants for Appropriated T Cell Responses in Virus-Like Particle-Based Vaccines. Front. Immunol..

[76] Jia R., Guo J.H., Fan M.W. (2012). The effect of antigen size on the immunogenicity of antigen presenting cell targeted DNA vaccine. Int. Immunopharmacol..

[77] Pape K.A., Catron D.M., Itano A.A., Jenkins M.K. (2007). The Humoral Immune Response Is Initiated in Lymph Nodes by B Cells that Acquire Soluble Antigen Directly in the Follicles. Immunity.

[78] Manolova V., Flace A., Bauer M., Schwarz K., Saudan P., Bachmann M.F. (2008). Nanoparticles target distinct dendritic cell populations according to their size. Eur. J. Immunol..

[79] Matloubian M., Clingan J.M. (2014). B cell-intrinsic TLR7 signalling is required for optimal B cell responses during chronic viral infection. J. Immunol..

[80] Hua Z., Hou B. (2013). TLR signaling in B-cell development and activation. Cell. Mol. Immunol..

[81] Eckl-Dorna J., Batista F.D. (2009). BCR-mediated uptake of antigen linked to TLR9 ligand stimulates B-cell proliferation and antigen-specific plasma cell formation. Blood.

[82] Jegerlehner A., Maurer P., Bessa J., Hinton H.J., Kopf M., Bachmann M.F. (2007). TLR9 Signaling in B Cells Determines Class Switch Recombination to IgG2a. J. Immunol..

[83] Krueger C.C., Thoms F., Keller E., Leoratti F.M.S., Vogel M., Bachmann M.F. (2019). RNA and Toll-Like Receptor 7 License the Generation of Superior Secondary Plasma Cells at Multiple Levels in a B Cell Intrinsic Fashion. Front. Immunol..

